# Habituation of parasympathetic-mediated heart rate responses to recurring acoustic startle

**DOI:** 10.3389/fpsyg.2014.01288

**Published:** 2014-11-20

**Authors:** Kuan-Hua Chen, Nazan Aksan, Steven W. Anderson, Amanda Grafft, Mark W. Chapleau

**Affiliations:** ^1^Department of Neurology, Carver College of Medicine, University of IowaIowa City, IA, USA; ^2^Neuroscience Graduate Program, University of IowaIowa City, IA, USA; ^3^University of Iowa Children’s HospitalIowa City, IA, USA; ^4^Department of Internal Medicine, Carver College of Medicine, University of IowaIowa City, IA, USA; ^5^Department of Molecular Physiology and Biophysics, University of IowaIowa City, IA, USA; ^6^Veterans Affairs Medical CenterIowa City, IA, USA

**Keywords:** acoustic startle responses, parasympathetic activity, heart rate variability, autonomic nervous system, stress, children, emotion regulation, startle habituation

## Abstract

Startle habituation is a type of implicit and automatic emotion regulation. Diminished startle habituation is linked to several psychiatric or neurological disorders. Most previous studies quantified startle habituation by assessing skin conductance response (SCR; reflecting sympathetic-mediated sweating), eye-blink reflex, or motor response. The habituation of parasympathetic-mediated heart rate responses to recurrent startle stimuli is not well understood. A variety of methods and metrics have been used to quantify parasympathetic activity and its effects on the heart. We hypothesized that these different measures reflect unique psychological and physiological processes that may habituate differently during repeated startle stimuli. We measured cardiac inter-beat intervals (IBIs) to recurring acoustic startle probes in 75 eight year old children. Eight acoustic stimuli of 500 ms duration were introduced at intervals of 15–25 s. Indices of parasympathetic effect included: (1) the initial rapid decrease in IBI post-startle mediated by parasympathetic inhibition (PI); (2) the subsequent IBI recovery mediated by parasympathetic reactivation (PR); (3) rapid, beat-to-beat heart rate variability (HRV) measured from the first seven IBIs following each startle probe. SCR and motor responses to startle were also measured. Results showed that habituation of PR (IBI recovery and overshoot) and SCRs were rapid and robust. In addition, changes in PR and SCR were significantly correlated. In contrast, habituation of PI (the initial decrease in IBI) was slower and relatively modest. Measurement of rapid HRV provided an index reflecting the combination of PI and PR. We conclude that different measures of parasympathetic-mediated heart rate responses to repeated startle probes habituate in a differential manner.

## INTRODUCTION

Brief aversive, acoustic stimuli trigger startle responses. Repeated exposures to the same stimuli further elicit “startle habituation,” meaning a reduction of behavioral and psychophysiological responses to the repeated startle stimuli (Turpin and Siddle, 1978a; Mata et al., 2009). Startle habituation is a type of implicit and automatic emotion regulation (Gyurak and Etkin, 2014) and the magnitude of startle response (i.e., eye-blink reflex) has a long history of use as a measure of defensive motivation and physiological index of fear (Bradley et al., 1999).

Recent research suggests that the magnitude of startle responses is meaningfully related to normative variation in adulthood personality and childhood temperament. For example, faster startle habituation has been noted in those who were high in extraversion and sensation seeking in college populations (LaRowe et al., 2006). In contrast, slower startle habituation has been linked to temperamental fearfulness or behavioral inhibition in both child (Quevedo et al., 2010; Barker et al., 2014) and adolescent samples (Reeb-Sutherland et al., 2009).

In clinical settings, diminished startle habituation has been repeatedly observed in patients with psychiatric or neurological disorders including schizophrenia, anxiety disorders, and Parkinson’s disease (Lader and Wing, 1964; Messina et al., 1972; Raskin, 1975; Geyer and Braff, 1982; Roth et al., 1990; Rothbaum et al., 2001; Nieuwenhuijzen et al., 2006). Magnitude of startle reflexes has also been useful in distinguishing children with anxiety disorders from controls (Waters et al., 2008) and adolescent males with conduct disorder from controls (Fairchild et al., 2008).

A better understanding of the determinants of the magnitude and rate of habituation to recurring startle stimuli may not only shed light on behavioral, cognitive, and emotional difficulty characteristic of several psychiatric and neurological disorders (Lader and Wing, 1964; Messina et al., 1972; Raskin, 1975; Geyer and Braff, 1982; Roth et al., 1990; Rothbaum et al., 2001; Nieuwenhuijzen et al., 2006) but also facilitate diagnoses and interventions for those conditions. Most previous studies in startle habituation have exclusively focused on examining changes in the electrodermal skin conductance response (SCR; reflecting sympathetic-mediated sweating; Lader and Wing, 1964; Raskin, 1975; Roth et al., 1990; Rothbaum et al., 2001), eye-blink reflex (Penders and Delwaide, 1971; Messina et al., 1972), and behavioral motor response (Nieuwenhuijzen et al., 2006). The habituation of parasympathetic effect to recurrent startle stimuli is not well understood. Given the strong relationship between parasympathetic activity and psychological and behavioral well-being (Porges et al., 1994; Porges, 2011; Thayer et al., 2012), we felt it was important to investigate the rate of habituation of parasympathetic-mediated heart rate (HR) responses to acoustic startle.

Startle stimuli evokes well-characterized tri-phasic changes in HR [inter-beat intervals (IBIs)], including (1) a rapid, transient decrease in IBI, followed by (2) an increase in IBI within a few seconds, and (3) a delayed decrease in IBI occurring over 20–60 s that dissipates over time (Davis et al., 1955; Graham and Clifton, 1966; Fernández and Vila, 1989; Reyes del Paso et al., 1993, 1994; Vila et al., 2007). In the present study, we focus on the first two components because they are primarily driven by the inhibition and reactivation (recovery) of parasympathetic nerve activity, respectively (in contrast, the third component involves increased sympathetic nerve activity). We refer to the first component as “*parasympathetic inhibition* (*PI*),” and to the second component as “*parasympathetic reactivation (PR).*”

Although habituation of PI and PR has been described previously, the rate and magnitude of habituation have been controversial (Vila et al., 2007). More specifically, early results suggested that PI habituates to a less extent than PR, but the difference was not statistically significant (Davis et al., 1955; Lang and Hnatiow, 1962; Graham and Clifton, 1966). In addition, PI was not always observed in previous studies (Graham and Clifton, 1966). In the present study, we re-visited this issue in a group of normally developing eight year old children.

An advantage of studying eight year old children is their homogeneity in terms of age, health, education, degree of socialization, and other factors that could confound the effect of parasympathetic modulation on HR response during emotion regulation (Byrne et al., 1996; Berntson et al., 1997; Bar-Haim et al., 2000; Hinnant et al., 2011). Previous research suggests that by age eight vagal modulation of HR has matured increasing the likelihood that findings from the current study may generalize to adult populations (Byrne et al., 1996; Berntson et al., 1997; Bar-Haim et al., 2000; Hinnant et al., 2011). In addition, many studies indicate that development of and variation in fear circuitry is relevant to both concurrent and future risk for psychopathology in both internalizing and externalizing spectrum (Degnan et al., 2010). A better understanding of the effect of parasympathetic modulation on HR responses during startle habituation in this age group can help shed light on fear circuitry and emotion regulation.

More recently, heart rate variability (HRV) became a popular measure for quantifying parasympathetic effect on human subjects (Malik et al., 1996; Berntson et al., 1997). HRV can be analyzed from IBIs using either time or frequency domain approaches (Malik et al., 1996; Chapleau and Sabharwal, 2011). In a previous study, Jovanovic and her colleagues examined trial-by-trial changes in HRV during startle habituation. They performed spectral analyses (frequency domain) on 10 s samples post-startle and reported no habituation in both post-traumatic stress disorder patients and healthy control subjects (Jovanovic et al., 2009). In the present study, we used a time-domain approach to re-address this issue. We measured rapid beat-by-beat HRV [root mean square of successive differences (RMSSDs)] from seven IBIs before and after each startle probe. The change/difference between RMSSD pre- and post-startle was then calculated and referred to as Δ*RMSSD7*. Habituation of ΔRMSSD7 was examined trial by trial. We used seven IBIs because preliminary analyses suggested that seven IBI covers the time course of both PI and PR post-startle.

The purpose of this study was to examine the habituation of parasympathetic effect on HR responses during recurring startle probes. Parasympathetic effect was quantified by three different measures: (1) PI, (2) PR, and (3) *Δ*RMSSD7. We hypothesized that those different measures reflect unique physiological and psychological processes that may habituate differently during repeated startle probes. To compare the three parasympathetic measures with typically used metrics, we also examined the habituation of SCR and motor response.

## MATERIALS AND METHODS

### PARTICIPANTS

Participants were 81 normally developing children recruited from the eastern Iowa area. They were eight years old at the time of evaluation. Due to procedural error or poor electrocardiogram (ECG) quality, six participants were excluded from data analyses. The remaining 75 participants included 33 boys and 42 girls. The participants are part of a long-term study investigating a variety of psychological and behavioral traits in addition to the physiological assessment (Kochanska and Kim, 2014; Kochanska et al., 2014).

### PROCEDURE

Research participation involved one laboratory visit. Parents consented and children provided assent in compliance with the policies of the University of Iowa Institutional Review Board. Children’s physiological responses were examined in five tasks presented in fixed order: rest one (3-min), deep breathing (2-min), startle (3-min), rest two (3-min), and anticipation (waiting for gift, 2-min). Only data from the startle task are presented in this report. Participants were seated in a comfortable chair facing a computer monitor. To prevent excessive motion during the startle task, participants were allowed to move around and readjust themselves before the startle task began. Participants and their parents were debriefed before they left the study.

### STARTLE TASK

**FIGURE 1:**
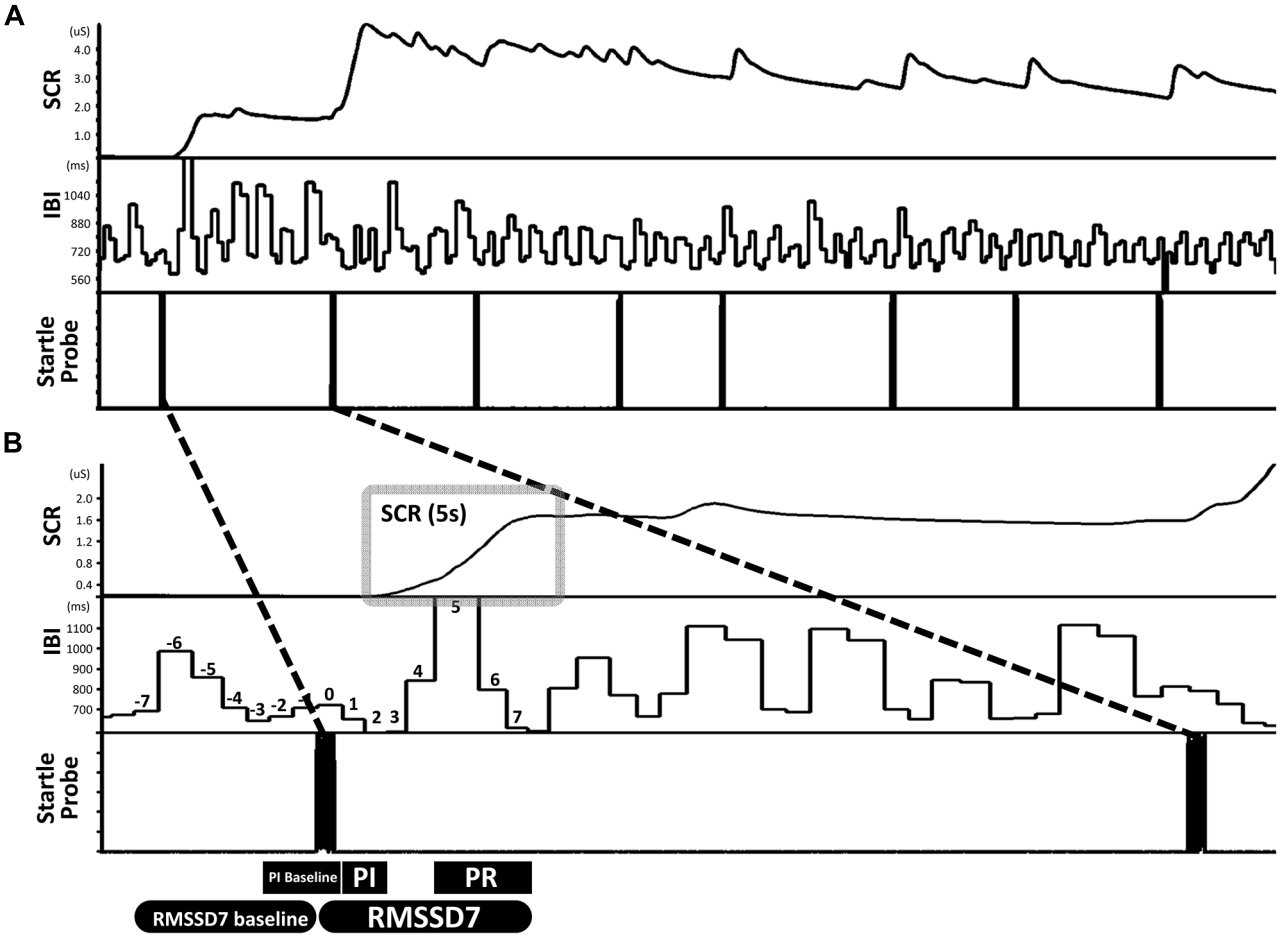
**An example illustrating the design of the startle task and the definitions of primary measures. (A)** The startle task consisted of eight startle probes. **(B)** Skin conductance response (SCR) was quantified as the area under curve in a 5-s window that begins at the inflection point of the increase from the immediately preceding stable baseline value. Parasympathetic inhibition (PI) was quantified as the change from baseline IBI pre-startle (mean of three IBIs) to the mean of the first two IBIs post-startle. Parasympathetic reactivation (PR) was quantified as the change from PI to the mean of the fifth through seventh IBIs post-startle. ΔRMSSD7 was calculated as the change in RMSSD from the seven IBIs pre-startle (baseline) to seven IBIs post-startle which included the IBI when the startle probe was delivered.

The participants were presented with eight startle probes (**Figure 1A**). The startle probe was an approximately 90 db white noise (8,192 Hz) lasting 500 ms, coming from two loudspeakers in front of the participants. Time between startle probes ranged from 15 to 25 s. In order to keep participant’s attention on the task, a series of abstract paintings (for examples, see **Figure S1**) were presented on the screen, changing at random intervals throughout the task. Participants were instructed to simply sit still and watch the pictures presented on a screen in front of them, and were told that they may hear some loud noises.

### DATA ACQUISITION AND DATA PROCESSING

#### Heart rate responses

Two foam electrodes were placed, one on the right side of the neck (close to right carotid artery) and the other on the left side of the abdomen, just below the rib cage. ECGs were recorded using a BIOPAC MP100 system at a sampling rate of 1000 Hz. HemoLab software (http://www.haraldstauss.com/HemoLab/HemoLab.php) was used to compute beat-to-beat IBIs from the ECG (R–R intervals). Artifacts in IBI data were corrected manually. For each startle probe, we calculated the following measures: (1) PI (representing the initial decrease in IBI), the difference between a pre-startle baseline (3 IBIs pre-startle, including the IBI when the startle probe was delivered) and the mean of the first two IBIs post-startle; (2) PR (representing the subsequent increase in IBI), the difference between the mean of the first two IBIs and the mean of the fifth through seventh IBIs post-startle. During PR, increases in IBI above baseline were also calculated (referred to as *reactivation overshoot*); (3) ΔRMSSD7, the change of RMSSD from a pre-startle baseline (consisting of seven IBIs) to the first seven IBIs following each startle probe including the IBI when the startle probe was delivered (**Figure 1B**).

#### Electrodermal activity: skin conductance response

The level of electrodermal activity was acquired by the BIOPAC MP100 system. Two foam electrodes were placed on the thenar and hypothenar eminences of the child’s left hand. Electrodermal data were recorded online at 1000 Hz and oﬄine down-sampled to 100 Hz before data processing. We analyzed electrodermal data from the children in which ECGs were analyzed (*n* = 75). Within these 75 children, six had poor electrodermal data quality and therefore were excluded before data processing. For the remaining 69 children, motion artifacts were identified by a trained research assistant (blinded to research hypotheses) and were manually corrected using Ledalab software (Benedek and Kaernbach, 2010). SCR induced by each startle probe was quantified as the change in conductance level measured over a 5-s time period beginning at the inflection point of the increase from the immediately preceding stable baseline value (area under curve, **Figure 1B**). To account for individual differences in general electrodermal reactivity, SCR was normalized by dividing by the range of each child’s skin conductance level over the whole testing session (Lykken and Venables, 1971).

#### Startle behaviors: motor responses

Participants were also video-recorded during the startle task. Due to variation in video recording quality, data from only 59 subjects could be coded for startle motor response by a trained research assistant (blind to the research hypotheses). Due to positioning of the camera, only whole-body startle responses could be coded which were defined as limb, trunk, and/or head movements evoked by the startle probe coded on a present/absence basis. The coder obtained inter-rater reliability for all judgments concerning startle-evoked movements (κ = 0.92) with one of the authors (Nazan Aksan).

### STATISTICAL ANALYSES

To determine if changes in HR responses and SCR were significant, we performed a set of planned one-sample *t*-tests, examining whether the *change* (before and after startle probe) was significantly higher than 0 (one-tailed). To examine if changes in HR responses and SCR from startle 2 to 8 significantly habituated from startle 1, we performed a set of planned paired *t*-tests, using the contrasts of startle 1 versus 2, startle 1 versus 3, etc. The Bonferroni method was used to correct *p* values for multiple comparisons (net *p* < 0.05).

Motor responses to startle were coded using binary codes (0 = no, 1 = yes). A set of McNemar *Chi-Square* tests were performed to determine if changes in the motor responses to recurring startle probes were significantly different from the response to the first startle probe. *p* values were corrected for multiple comparisons using the Bonferroni method (net *p* < 0.05).

To determine if changes in different psychophysiological measures correlated with each other and with motor responses to startle probes, a set of Pearson correlations were computed for the following measures: changes in (1) PI, (2) PR, (3) reactivation overshoot, (4) ΔRMSSD7, (5) SCR, and (6) motor responses from the first four to the last four startle probes. Statistical significance was considered when *p* < 0.05 (two-tailed test).

## RESULTS

### MOTOR RESPONSE AND SCR TO STARTLE PROBES

**FIGURE 2:**
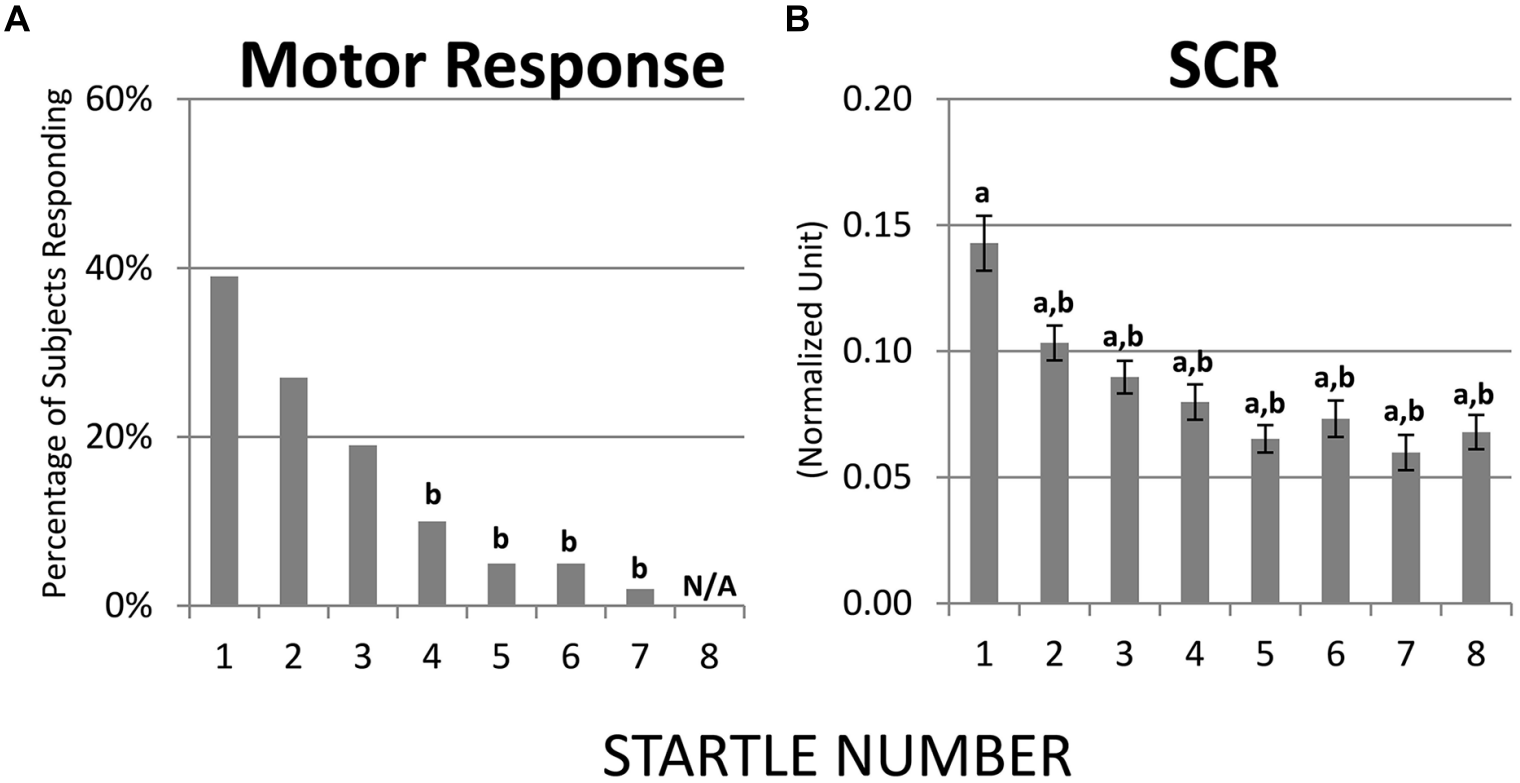
**Habituation of motor response and SCR over recurring startle probes. (A)** Compared to startle 1, startles 4–7 were associated with lower percentage of children showing a motor response; at startle 8 none of the children showed a motor response. **(B)** SCR habituated immediately at startle 2. a = significantly higher than baseline (0); b = significantly lower than the initial response (i.e., startle 1).

Habituation of motor response and SCR were both robust. Results from McNemar *Chi-Square* tests indicated that, compared to the first startle probe, the fourth through the seventh probes were associated with lower percentage of children showing a motor response and at the eighth probe none of the children showed a motor response (*p*s < 0.01, **Figure 2A**). Regarding SCR, one-sample *t*-tests revealed that the eight startle probes all elicited significant SCR [*t*s(68) > 8.60, *p*s < 0.01, **Figure 2B**]. Paired *t*-tests showed that compared to the first startle probe, SCRs were significantly lower at the second through the eight startle probes [*t*s(68) > 3.79, *p*s < 0.01, **Figure 2B**].

### CARDIAC IBI RESPONSES TO STARTLE PROBES

**FIGURE 3:**
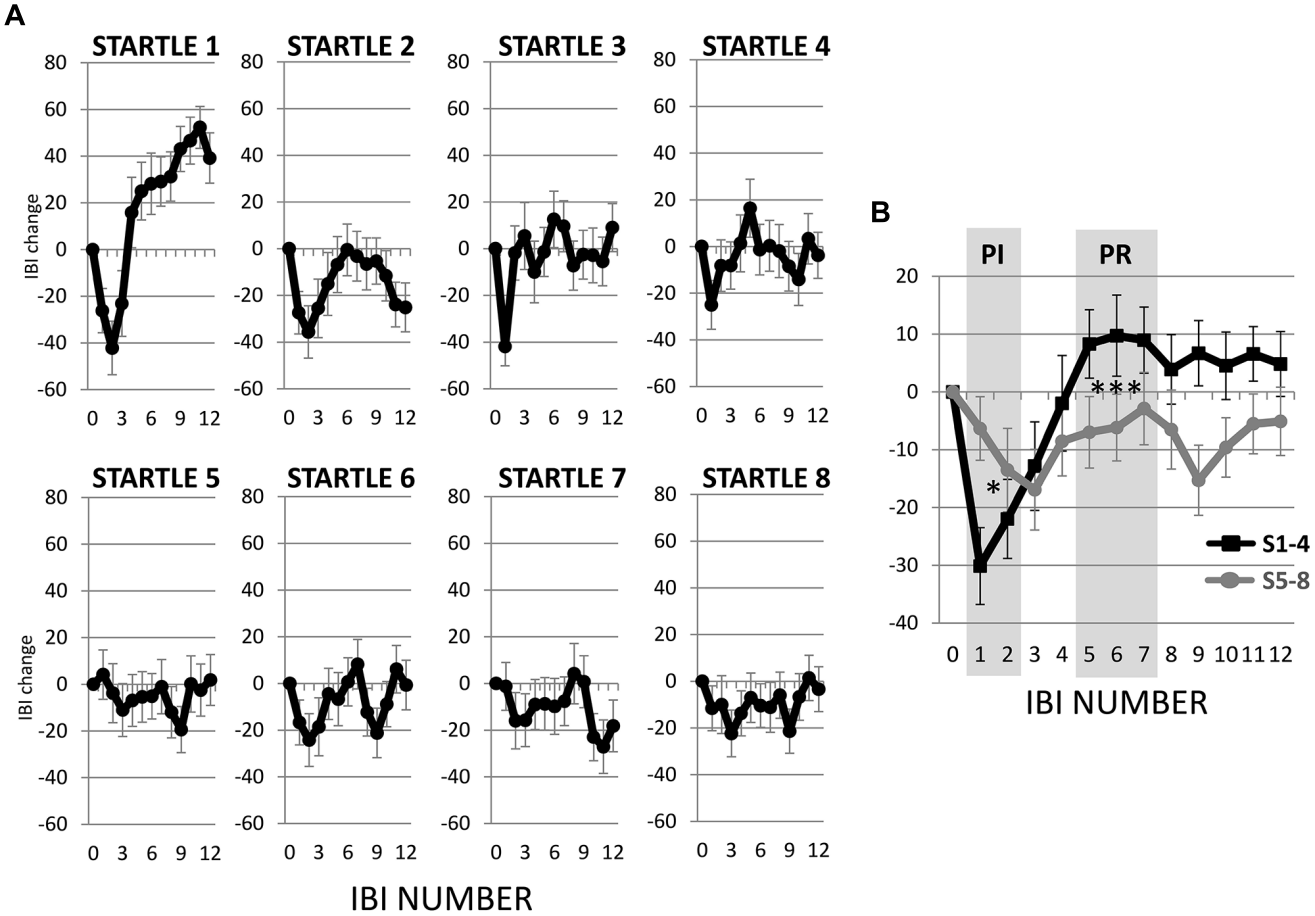
**Inter-beat interval (IBI) changes following startle probes. (A)** Startle probe 1 evoked a rapid, transient decrease in IBI (PI), which was followed by an increase in IBI (PR) that exceeded the baseline IBI (reactivation overshoot). Both PI and PR habituated over recurring startle probes. **(B)** Average of IBI changes from the first four versus the last four startle probes. Both PI and PR significantly decreased (*p*s < 0.05, **Table 2**). *p < 0.05; ***p < 0.001.

**FIGURE 4:**
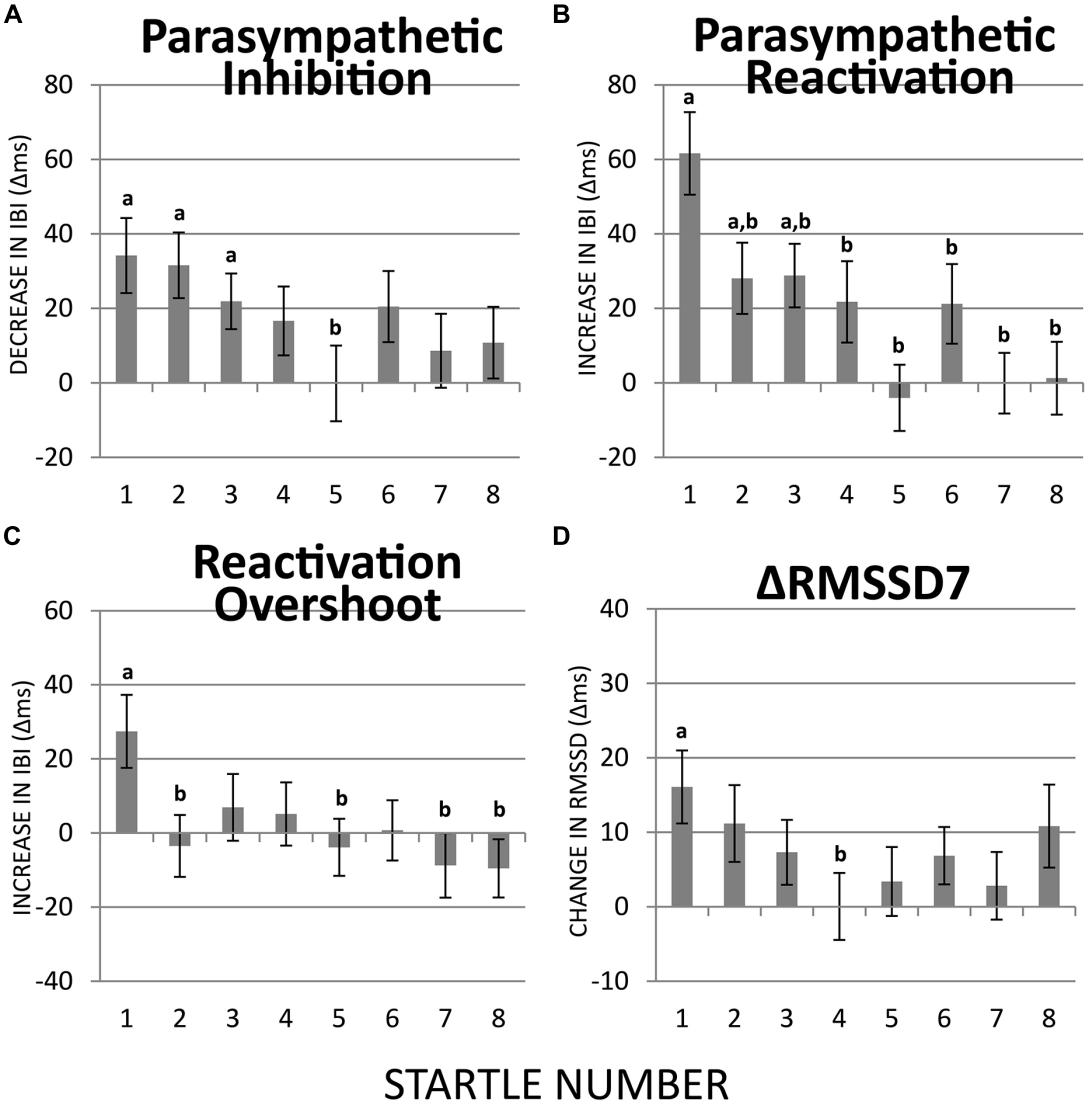
**Habituation of PI, PR, reactivation overshoot, and ΔRMSSD7 over recurring startle probes. (A)** PI was significant at startles 1–3 but not at the remaining five startle probes. PI at startle 5 was significantly less than at startle 1. **(B)** PR was significant at startles 1–3 but not at the remaining five startle probes. PR habituated at startle 2. **(C)** Reactivation overshoot was significant at startle 1; it habituated at startle 2. **(D)** The first startle probe triggered a significant increase in RMSSD7 from baseline, which habituated significantly at startle 4. a = significantly higher than baseline; b = significantly lower than at startle 1.

The first startle probe evoked a rapid, transient decrease in IBI followed by a recovery of IBI that exceeded the baseline IBI measured before the first startle probe was introduced (**Figure 3A**). Both the initial decrease in IBI and subsequent increase in IBI triggered by the first startle probe were statistically significant (**Figures 4A,B**). As mentioned above, we refer to the rapid decrease and increase in IBI as PI and PR, respectively (Graham and Clifton, 1966; Fernández and Vila, 1989; Reyes del Paso et al., 1993, 1994; Vila et al., 2007). In addition, we refer to the increase in IBI above baseline as “reactivation overshoot” (**Figure 4C**).

The startle-induced PI (decrease in IBI) was significant for the first three startle probes [*t*s(74) > 2.93, *p*s < 0.05], but was attenuated and no longer statistically significant for startles 4–8 (**Figure 4A**). Comparing with the first startle probe, only the fifth startle probe triggered a significantly less PI [*t*(74) > 2.70, *p* < 0.05].

Regarding PR (the subsequent increase in IBI), the first three startle probes triggered significant PR [*t*s(74) > 2.93, *p*s < 0.05; **Figure 4B**]. Paired *t*-tests revealed that PR was significantly less at startle probes 2–8 than at startle probe 1 [*t*s(74) > 2.70, *p*s < 0.05], which suggests a relatively more rapid habituation of PR than PI.

Reactivation overshoot was only significant at the first startle [*t*(74) > 2.79, *p* < 0.05]. Significant habituation (from the first startle) was observed at startle 2 [*t*(74) > 2.59, *p* < 0.05] as well as startles 5, 7, and 8 [*t*s(74) > 2.58, *p*s < 0.05; **Figure 4C**].

ΔRMSSD7 was significantly increased from the baseline value at startle 1 [*t*(74) > 3.28, *p* < 0.01], which gradually decreased at the startles 2 and 3, and became significantly smaller than startle 1 at startle 4 [*t*(74) > 2.52, *p* < 0.05; **Figure 4D**].

**Table 1** summarizes the results described above.

**Table 1:** Summary of significant responses to startles (S) and significant habituation from the first startle.

	Significant response (Response > 0/baseline^1^)	Significant habituated from Startle 1 (Response < S1^2^)
Parasympathetic inhibition (PI)	S1, S2, S3	S5
Parasympathetic reactivation (PR)	S1, S2, S3	S2, S3, S4, S5, S6, S7, S8
Reactivation overshoot	S1	S2, S5, S7, S8
ΔRMSSD7	S1	S4
Motor response	–	S4, S5, S6, S7, S8^3^
SCR	S1, S2, S3, S4, S5, S6, S7, S8	S2, S3, S4, S5, S6, S7, S8

*^1^Equals to note “a” in **Figures 2** and **4**; ^2^Equals to note “b” in **Figures 2** and **4**; ^3^No participant showed motor response to the startle probe.*

### BIVARIATE CORRELATIONS BETWEEN RESPONSES TO STARTLE

To determine if habituation in different psychophysiological measures correlated with each other and with motor responses to startle probes, we first computed the changes in PI, PR, reactivation overshoot, ΔRMSSD7, motor responses and SCR from the first four startle probes to the last four startle probes (**Table 2**). As shown in **Table 2** and **Figure 3B**, habituations of PI and PR were both significant when comparing the average of changes from the first four to the last four startle probes. We then examined the correlations among those measures. As shown in **Table 3**, habituation of PI and PR was strongly correlated (*r* = 0.55). The habituation of ΔRMSSD7 was significantly correlated with habituation of PR (*r* = 0.40), but not with habituation of PI (*r* = 0.15). Additionally, habituation of ΔRMSSD7 was significantly correlated with habituation of reactivation overshoot (*r* = 0.27). Habituation of SCR was significantly correlated with habituation of PR (*r* = 0.25) and was marginally correlated with habituation of the motor response (*r* = 0.23, *p* < 0.10).

**Table 2:** Mean ± SE of the primary measures, and comparisons between averaged response from the first 4 versus the last 4 startle probes.

	Startle 1–4	Startle 5–8	*t*
Parasympathetic inhibition (ms, PI)	26.06 ± 5.87	9.93 ± 5.74	2.32*
Parasympathetic reactivation (ms, PR)	35.05 ± 6.85	4.58 ± 6.00	4.42***
Reactivation overshoot (ms)	8.99 ± 5.21	–5.35 ± 4.82	2.18*
ΔRMSSD7 (ms)	8.65 ± 2.78	5.97 ± 2.22	0.79
Motor response (frequency)	0.95 ± 0.15	0.12 ± 0.06	6.16***
SCR (n.u.)	0.10 ± 0.01	0.07 ± 0.01	7.14***

n.u., normalized units. *p < .05, **p < .01, ***p < .001.

**Table 3:** Bivariate correlations among primary variables.

	Cardiac measures					
	Parasympathetic inhibition	Parasympathetic reactivation	Reactivation overshoot	ΔRMSSD7	Motor response	SCR
Parasympathetic inhibition (PI)	–					
Parasympathetic reactivation (PR)	0.55***	–				
Reactivation overshoot	–0.48***	0.47***	–			
ΔRMSSD7	0.15	0.40***	0.27*	–		
Motor response	0.20	0.18	–0.01	0.10	–	
SCR	0.15	0.25*	0.11	0.14	0.23^ϕ^	–

SCR, skin conductance response. ^ϕ^*p* < 0.10, *p < 0.05, ***p < 0.001.

## DISCUSSION

A variety of parasympathetic, sympathetic, and behavioral responses to recurrent acoustic startle probes were measured in a defined population of eight year old children in this study. The major findings were: (1) The first startle probe induced a significant motor response, SCR, and rapid and transient PI, followed by a PR and overshoot; (2) Habituation of the motor response and SCR was robust and consistent; (3) PR habituated relatively quickly, whereas PI habituated slower, (4) Habituation of ΔRMSSD7 was faster than habituation of PI but slower than habituation of PR; (5) Habituation of SCR was significantly correlated with habituation of PR and marginally correlated with habituation of motor response; (6) Habituation of ΔRMSSD7 was significantly correlated with habituation of PR, but not with habituation of PI. We conclude that different measures of parasympathetic-mediated HR responses habituate in a differential manner during exposure of children to recurrent startle probes.

We discuss below the results of previous studies relevant to our findings, reasons for differences in habituation among the parasympathetic metrics examined, implications for clinical settings, and limitations in the study design.

### PREVIOUS STUDIES OF HR RESPONSES TO STARTLE

Early results suggested that PI (the initial decrease in IBI following startle probes) was only evident in some studies. When PI occurred, it habituated to a less extent than PR (the subsequent increase in IBI; Davis et al., 1955; Lang and Hnatiow, 1962; Graham and Clifton, 1966). In the present study, we observed significant PI. Our results also confirm that the habituation of PI was less compared with PR. Of all the parasympathetic metrics we examined, PR showed the greatest habituation which was in part the result of strong overshoot of the increase in IBI at the first startle.

Recent studies provided evidence that low HRV measured during a resting baseline period is associated with decreased startle habituation of the eye-blink response (Gorka et al., 2013a,b). To our knowledge, except for the Jovanovic et al. (2009) study, no other studies have attempted to quantify HRV *during* the process of startle habituation. In this study, we used time-domain based ΔRMSSD7 as an easy-to-measure index of parasympathetic modulated HRV. Our results demonstrate that the first startle probe elicited a significant increase in RMSSD7 from baseline, which gradually decreased at startles 2 and 3, and became significantly smaller at startle 4 compared with startle 1. ΔRMSSD7 during the last four startle probes were less stable, showing a less discernable pattern compared with earlier probes.

### REASONS FOR DIFFERENCES IN HABITUATION AMONG PARASYMPATHETIC METRICS

Our results demonstrated that PR showed a rapid and strong habituation after the first startle. In contrast, habituation of PI was slower and relatively modest. We reason this could be a result of the different underlying mechanisms. More specifically, the magnitude of PI (decrease in IBI) immediately post-startle is determined in part by the cardiac vagal tone present during the baseline period prior to startle. In contrast, the subsequent PR (increase in IBI) is dependent on both the magnitude of the initial inhibition and the additional PR and overshoot.

ΔRMSSD7 provides an integrative measure of both inhibitory and reactivating components of parasympathetic modulation. The habituation of ΔRMSSD7 showed a pattern in between PI and PR. ΔRMSSD7 at startle 4 was significantly less than ΔRMSSD7 at startle 1. This habituation was slower than the habituation of PR but faster than the habituation of PI. In addition, ΔRMSSD7 was no longer significantly greater than baseline (measured from 7 IBIs before each startle probe) for startles 2–8, which illustrates the rapid habituation.

### IMPLICATIONS FOR CLINICAL SETTINGS

Both PI and PR habituated to recurrent startle probes but at different rates. This result implies that there may have been different mechanisms involved for the individuals to adapt for recurrent aversive environmental stimuli, and those mechanisms may have different time courses for effect.

It has been suggested that PI (decrease in IBI) immediately post-startle reflects a defensive reflex, while PR (increase in IBI) reflects an orienting reflex (Graham and Clifton, 1966). As the task progresses with recurring startles, one might expect a decrease in novelty associated with orienting responses, and an increase in expectation associated with more-controlled defensive responses (comparing to the initial automatic defensive reflex), possibly resulting in decreases in both PI and PR.

Decreases in PI and PR may also reflect the transition from a “passive coping” to an “active coping” phase. According to Obrist (1976), HR deceleration reflects a process of “passive coping,” meaning that the heart is passively influenced by the vagus nerve activity rather than by top–down cognitive effort exerted by the individual. “Passive coping” occurs when an aversive stimulus has just been encountered and before the individual has prepared a response to it (Obrist, 1976). In the current study, the high level of PR in the first few startle probes may reflect the process of “passive coping” as participants had little knowledge of what the ‘loud sounds’ would be like and how frequently they would be delivered. After the individual has encountered the aversive stimuli a few times, he or she may build expectations in the “active coping” phase, a more controlled cognitive process which is associated with increased sympathetic activity, resulting in HR acceleration (Obrist, 1976). In the current study, following repeated exposures to the startle probe, we observed less PR consistent with “active coping”.

Results from this study have significant implications for clinical settings. Understanding changes in parasympathetic effect during startle habituation may shed light on both passive and active emotion regulatory processes during sustained exposure to aversive events. Previous studies have examined parasympathetic effect in responding to environmental changes, such as when the individual moves from rest to a stressful condition or vice versa (i.e., vagal withdraw and recovery, respectively; Porges et al., 1994; Santucci et al., 2008; Gentzler et al., 2009). The findings from the present study speak to this mechanism in a sustained aversive event. This coping process is likely to be automatic and implicit. However, it may require the individual to actively encode the intensity and the timing of the aversive stimuli, and anticipate future events, including the probability, timing, and possible outcomes.

A substantial number of studies have found reduced habituation in sympathetic-mediated and motor responses to recurrent startle probes in several psychiatric and neurological disorders, including schizophrenia, anxiety disorders, and Parkinson’s disease (Lader and Wing, 1964; Raskin, 1975; Geyer and Braff, 1982; Roth et al., 1990; Rothbaum et al., 2001; Jovanovic et al., 2009, 2010). Results from the present study demonstrated a significant correlation between habituation of PR and habituation of the sympathetic-mediated SCR. This finding suggests that patients with the above psychiatric/neurological disorders may also exhibit impaired parasympathetic modulation during sustained aversive conditions. We suggest future studies to directly examine the function/dysfunction of parasympathetic modulation during sustained aversive conditions in those clinical populations.

### LIMITATIONS IN STUDY DESIGN

There are several limitations in our study to consider. The present study did not control for the depth or frequency of breathing, which could significantly affect the changes in IBI induced by acoustic startle (Turpin and Siddle, 1978b). More specifically, individuals with deeper breathing may exhibit increased HRV than individuals with shallower breathing. In addition, previous studies have found that acoustic startle probes induce a rapid deep breath or gasp in some subjects and that this deep breath may contribute to the initial decrease in IBI post-startle (Smith and Strawbridge, 1969; Hart, 1975; Harver and Kotses, 1987; Reyes del Paso and Vila, 1993). Although we did not systematically analyze the effect of respiration, we did compare a subgroup of children (*n* = 12) who showed exaggerated inspiration post-startle with a subgroup of children whose respiration did not appear to be altered (*n* = 14). Analysis of the IBI responses to the first two startle probes suggests that children exhibiting deeper inhalations post-startle show a trend of greater PI and greater PR than children with unchanged respiration. The between-group difference in PR was significant for startle probe 2 [*t*(24) = 2.51, *p* = 0.019], while the difference in PR was marginally significant for startle probe 1 [*t*(24) = 1.84, *p* = 0.078].

Many startle habituation studies, including the present study, have used relatively short intervals between startle probes (15–25 s; Geyer and Braff, 1982; Jovanovic et al., 2009), while others have used longer intervals (>45 s; Lacey and Smith, 1954; Davis et al., 1955; Lader and Wing, 1964; Raskin, 1975; Rothbaum et al., 2001). One advantage of our design is that use of short intervals facilitates the rate of habituation. However, this design may not allow sufficient time for parasympathetic effect on HR to fully return to the original baseline levels. To test this possibility, we compared baseline RMSSD7 (the index of parasympathetic modulation) before each of eight startle probes. Baseline RMSSD7 did not significantly differ over the period of the first seven startle probes (**Figure S2**). The results suggest that the inter-startle intervals of our study were sufficient for parasympathetic modulation to return to baseline levels prior to the next startle probe.

We also considered that our finding that RMSSD7 increased from baseline at startle 1 and habituated gradually may have been confounded by a possible change in mean HR during the task. Previous studies have demonstrated that changes in HR *per se* can dramatically affect indices of HRV independent of changes in cardiac autonomic regulation (Sacha and Pluta, 2005; Billman, 2013). To address this issue, we compared the means of the IBIs used to calculate the first ΔRMSSD7 (IBIs pre- versus post-startle probe 1) and found no significant difference [*t*(74) = 0.03, *p* = 0.98], which suggested that the increase in RMSSD7 triggered by the first startle probe was not confounded. We also analyzed our data (trial-by-trial habituation in ΔRMSSD7) after correcting for changes in mean IBI as the task progressed (Sacha and Pluta, 2005; Billman, 2013), and the results confirmed our conclusions.

## SUMMARY

In summary, we have demonstrated that different measures of parasympathetic modulation of HR responses habituate differentially in children exposed to recurrent startle probes. PR, following the initial PI, shows the most rapid and robust habituation of all of the measures. Our results are consistent with “active coping” hypothesis that has been proposed in adapting to sustained aversive events. We introduced ΔRMSSD7, a simple measure which captures the effects of both PI and PR and demonstrated that it is a valid measure of quantifying parasympathetic modulation during startle habituation. The current findings extend the existing literature on startle responses beyond the motor and sympathetic systems, and inform differences in various measures of parasympathetic modulation. These findings are well-situated to inform future studies that attempt to specify multivariate response profiles in startle paradigms that may come to serve as endo-phenotypes for psychopathology in both normative and non-normative samples.

## Conflict of Interest Statement

The authors declare that the research was conducted in the absence of any commercial or financial relationships that could be construed as a potential conflict of interest.

## Supplementary material

Figure S1 **Examples of the abstract paintings used in the startle task.** In order to keep participant’s attention on the task, a series of abstract paintings were presented on the screen throughout the task.

Figure S2 **Baseline RMSSD7 (measured from seven IBIs prior to each startle probe) did not change significantly until the last startle probe.** a = significantly different from the previous startle probe (two-tailed tests, *p* value corrected using the Bonferroni method).
