# Supplementary figures and images for: Habituation of parasympathetic-mediated heart rate responses to recurring acoustic startle
Source: Front Psychol. 2014 Nov 20;5:1288. doi: 10.3389/fpsyg.2014.01288 (PMC4238409; doi:10.3389/fpsyg.2014.01288)

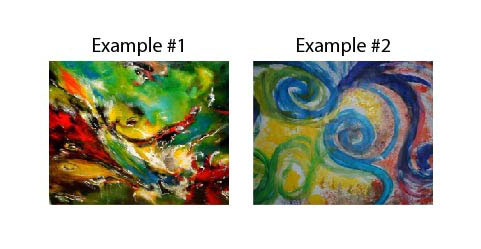

Supplement: Figure S1 — Examples of the abstract paintings used in the startle task. In order to keep participant’s attention on the task, a series of abstract paintings were presented on the screen throughout the task. [file Image_1.JPEG]

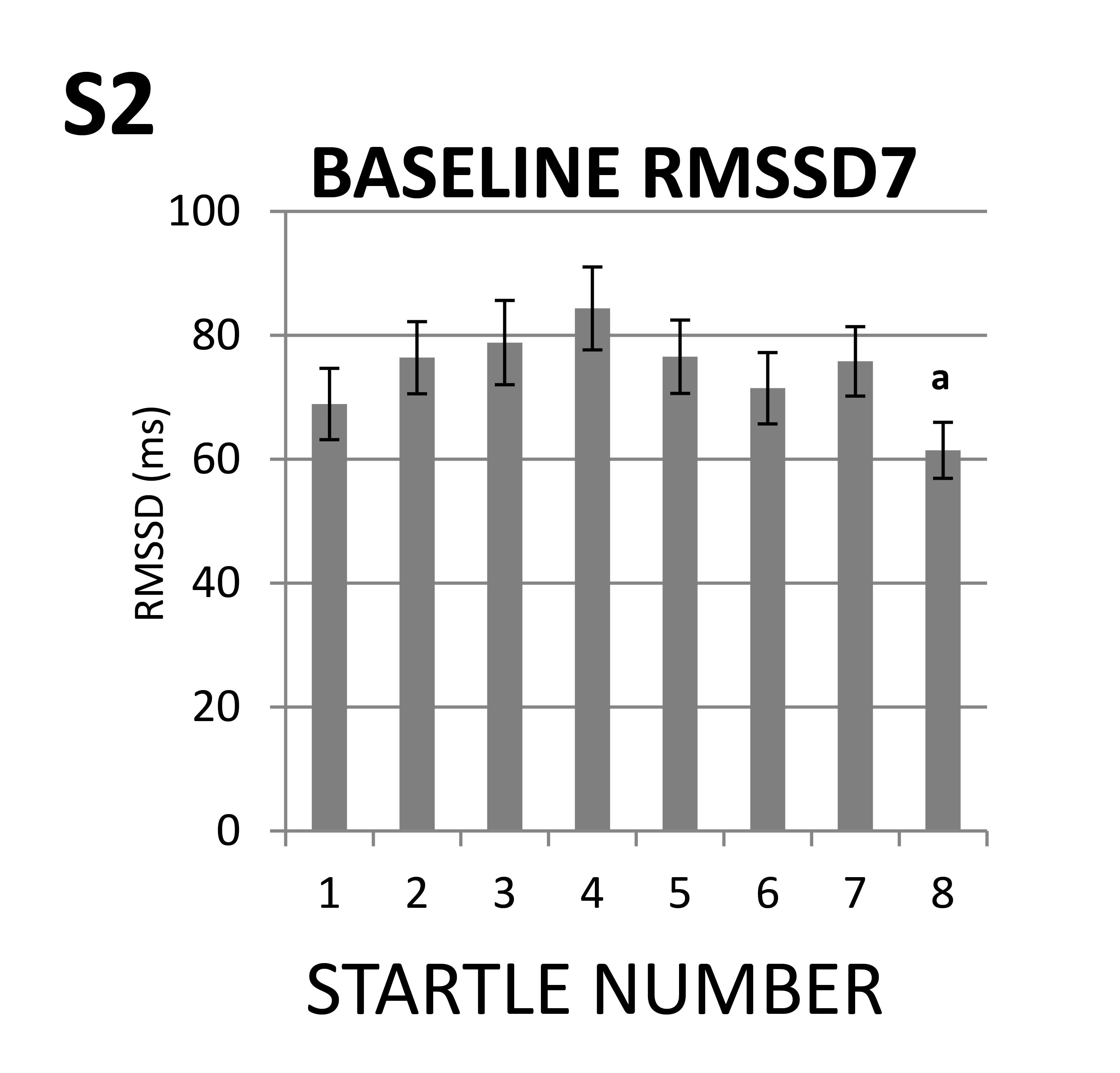

Supplement: Figure S2 — Baseline RMSSD7 (measured from seven IBIs prior to each startle probe) did not change significantly until the last startle probe. a = significantly different from the previous startle probe (two-tailed tests, p value corrected using the Bonferroni method). [file Image_2.JPEG]
